# Examination of postmortem changes in the lungs, trachea, and bronchi in a rat model imaged with small-animal computed tomography

**DOI:** 10.20407/fmj.2022-002

**Published:** 2022-07-22

**Authors:** Takahiro Matsuyama, Seiichiro Ota, Yoshitaka Inui, Naoko Fujii, Tetsuya Tsukamoto, Ichiro Isobe, Katsumi Tsujioka, Shizuko Nagao, Ryosuke Tanabe, Hiroshi Toyama

**Affiliations:** 1 Department of Radiology, Fujita Health University, School of Medicine, Toyoake, Aichi, Japan; 2 Department of Radiology, Fujita Health University Okazaki Medical Center, Okazaki, Aichi, Japan; 3 Department of Diagnostic Pathology, Fujita Health University, School of Medicine, Toyoake, Aichi, Japan; 4 Department of Legal Medicine, Fujita Health University, School of Medicine, Toyoake, Aichi, Japan; 5 Faculty of Radiological Technology, Fujita Health University, School of Medical Science, Toyoake, Aichi, Japan; 6 Center for Clinical Trial and Research Support, Fujita Health University, Toyoake, Aichi, Japan; 7 Tanabe Clinic, Matsuyama, Ehime, Japan

**Keywords:** Computed tomography, Trachea volume, Postmortem change, Forensic radiology

## Abstract

**Objectives:**

As less autopsies are performed, the need for postmortem computed tomography (PMCT) as an alternative is increasing. It is important to know how postmortem changes over time are reflected on CT, in order to improve the diagnostic capability of PMCT and replace forensic pathology evaluations such as time of death estimation.

**Methods:**

In this study, we examined temporal changes on postmortem chest CT images of a rat model. After acquiring antemortem images under isoflurane inhalation anesthesia, the rats were euthanized with a rapid intravenous injection of anesthetics. From immediately after death to 48 hours postmortem, chest images were acquired using small-animal CT. The 3D images were then evaluated on a workstation to measure the antemortem and postmortem air content in the lungs, trachea, and bronchi over time.

**Results:**

The air content in the lungs decreased, but the air content of the trachea and bronchi temporarily increased 1–12 hours postmortem, then decreased at 48 hours postmortem. Therefore, the measurement of trachea and bronchi volumes on PMCT could be an objective way to estimate the time of death.

**Conclusions:**

While the air content of the lungs decreased, the volume of the trachea and bronchi temporarily increased after death, indicating the potential to use such measurements to estimate time of death.

## 1. Introduction

In Japan, postmortem computed tomography (PMCT) is more widely used than autopsy.^1^ Since 1985, PMCT has been used in situations where performing an autopsy would be difficult, and also to determine the need for an autopsy.^1^

PMCT is now routinely used in many sites worldwide,^2^ and as the use of autopsy is decreasing globally,^3^ the need for and frequency of use of postmortem imaging as an alternative or concomitant tool is increasing.^4^ As such, many scientific articles on PMCT have been published in recent years.^2,5,6^ In 2003, the Japanese Society of Autopsy Imaging was established, and it is estimated that more than 20 000 PMCTs are now performed each year.^7^ PMCT, which can dramatically improve the quality and effectiveness of an autopsy, is a noninvasive, rapid, and objective procedure, clarifying characteristics that may not be detected on conventional autopsy. It also results in permanent and accessible data that can be examined using 3D image processing.^8^

However, PMCT is subject to several limitations, such as a lack of differentiation between normal postmortem changes and pathological changes, no diagnostic ability for cardiac death, and artifacts in the images. As postmortem biology differs from that in the antemortem state, antemortem CT (AMCT) and PMCT findings also differ, which sometimes makes it difficult to differentiate normal postmortem change from pathological change.^9–11^ Once the typical imaging findings of postmortem changes are clarified, it should be easier to determine whether imaging findings on PMCT represent normal postmortem changes or changes associated with the cause of death, which should improve the ability to diagnose the cause of death using PMCT.

In this study, we evaluated the use of chest PMCT for examination of the respiratory system. In respiratory-related causes of death, such as drowning, it may be difficult to diagnose the cause of death using chest PMCT.^12^ Recognition of postmortem changes and accurate interpretation are important for determining the cause of death.^12^

In forensic pathology, the volume, weight, air content of the lungs, and pleural fluid are important indices used to examine the cause of death, and can provide information relevant to the autopsy findings.^13,14^ The temporal courses of pleural effusion and lung aeration can be predicted, but there are no reports on the quantitative evaluation of temporal changes in the lungs and airway system using PMCT. Therefore, we examined these changes in this study, excluding other forensic pathology indices.

Generally, when PMCT is performed to determine the cause of death for forensic purposes, it is not usually possible to compare the results with AMCT unless the patient died during hospitalization. Furthermore, in most cases, only one postmortem image is taken, making temporal comparisons impossible. Therefore, it can be difficult to estimate the time of death and determine whether PMCT findings reflect postmortem changes or are related to the cause of death.

In this study, we used a rat model to acquire CT before death, immediately after death (0 hours), and over time up to 48 hours after death. The use of an animal model allows temporal and frequent PMCT imaging, which is difficult to perform on humans. Furthermore, our model provided the advantages of knowing the cause and time of death.

## 2. Methods

### 2.1. Imaging

After obtaining approval from the Institutional Animal Care and Use Committee at Fujita Health University, we conducted the experiments following the protocol of the university’s Center for Clinical Trials and Research Support. Rats (weight 300–400 g, 9–10-week-old, wild-type, SPF Wistar, male, n=9) were housed at the Center for Clinical Trials and Research Support with a 12:12 light−dark cycle, temperature of 23°C±3°C, humidity of 50%±10%, and 12 ventilations/hour. A small-animal CT scanner was used for the imaging (Micro X-ray CT scanner for animal experiment, Rigaku Corporation, Tokyo, Japan). The tube voltage was 90 kV, tube current 80 μA, and field of view 60 mm.

The rats were put under 1% isoflurane inhalation anesthesia and an intravenous line was secured in the tail vein using a 22G indwelling needle. The rats were then fixed to the imaging stage in a prone position using non-woven tape. First, an antemortem chest CT image was acquired under 1% isoflurane inhalation anesthesia. Immediately after this acquisition, 1.0 mL of pentobarbital sodium was rapidly injected through the intravenous line to euthanize the rat. Chest CT was then acquired immediately after euthanasia and at 1, 2, 3, 6, 9, 24, and 48 hours later, with the rat in the same position and maintained under the same conditions as in the animal holding room.

### 2.2. Imaging and histopathological analysis

All data were stored in DICOM format and Workstation 3D (AZE Virtual Place Raijin, Canon Medical Systems Corporation, Otawara, Tochigi, Japan) was used for the image analysis. Using an automatic lung analysis function equipped with manual adjustment, the aerated lungs, trachea, and bronchi were identified. The analyzed range of the trachea and bronchi was between the upper end of the sternum to the base of the lungs. Measurements were made of the antemortem and postmortem air content of the lungs, trachea, and bronchi over time using 3D images.

Temporal changes in the air content of the lungs, trachea, and bronchi were compared using Friedman’s test.

Immediately after CT imaging, thoracotomy was performed and after removing both lungs, trachea, and bronchi, formalin solution was injected into the trachea to fix the sample. The excised lungs, trachea, and bronchi were then fixed with paraffin, stained with hematoxylin and eosin, and organic changes were assessed.

## 3. Results

**Figure 1:**
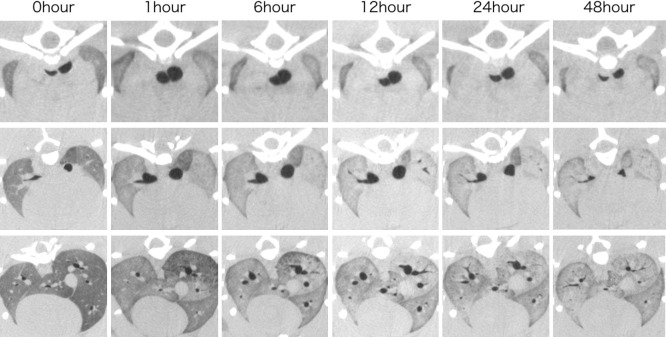
Cross-sectional computed tomography (CT) scan The top image shows the level of tracheal bifurcation, the middle image shows the level of subtracheal bifurcation, and the bottom image shows the base of the lung. Compared with 0 hours, the bronchi expanded 1–12 hours postmortem, then shrank after 24 hours. After 48 hours, the air content decreased and pulmonary edema and congestion were confirmed.

**Figure 2:**
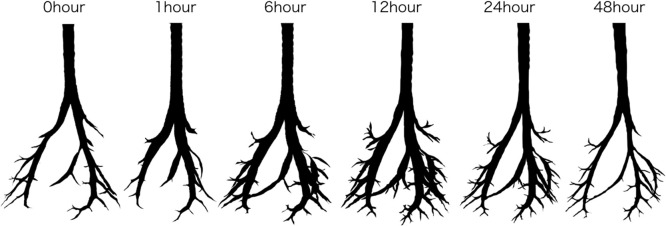
3D image analysis identifies only the trachea and bronchi Compared with 0 hours, the left and right bronchi are thicker at 12 hours postmortem, and peripheral bronchi are clearly identifiable. The air contents of the trachea and bronchi were: 0.2745, 0.3009, 0.3538, 0.5079, 0.4504, and 0.2397 mL at 0, 1, 6, 12, 24, and 48 hours postmortem, respectively.

**Figure 3:**
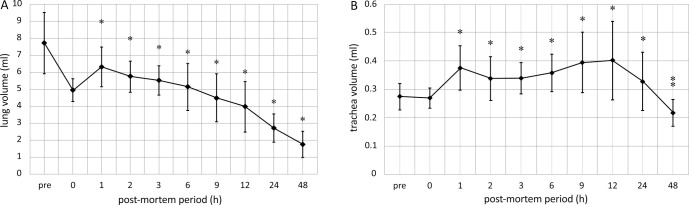
Air content in the lungs, trachea, and bronchi over time (A) Lungs and (B) trachea and bronchi The air content of the lungs decreased significantly after death (*P<0.05), whereas the air content of the trachea and bronchi increased significantly 1–12 hours postmortem compared with 0 hours (*P<0.05). After 48 hours, the air content significantly decreased (**P<0.05).

Comparisons with the CT images taken before and immediately after euthanasia (0 hours) revealed that the air content of the lung fields generally decreased with the passage of time after death. However, the trachea and bronchi temporarily expanded (Figures 1 and 2). The mean air content (mL) of the lungs showed an immediate and considerable decrease at 0 hours, then a continuous decrease over time (Figure 3A). The mean air content (mL) of the trachea and bronchi increased remarkably 1–24 hours postmortem, and then decreased 48 hours later (Figure 3B).

**Figure 4:**
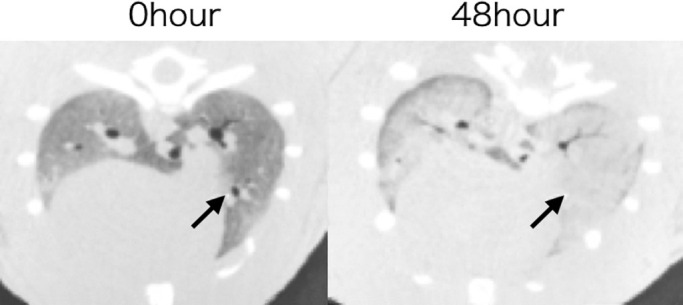
Fluid in the trachea and bronchi The bronchus on the ventral side of the right lung (arrow) shows loose air due to fluid accumulation 48 hours postmortem.

PMCT images taken 48 hours after death confirmed lung fluid and congestion. Furthermore, some bronchi lost air content because of fluid collection (Figure 4). Histopathological analysis confirmed the air content decrease, with edema and congestion being present. However, no clear organic postmortem changes were indicated in the trachea and bronchi from 0 to 48 hours postmortem.

## 4. Discussion

The study results show that lung volume temporarily decreased after death, whereas tracheal and bronchial volumes temporarily increased. Currently, we are not aware of any report on postmortem changes in lung, tracheal, or bronchial volume measured using PMCT.

The human pupil immediately dilates after death, then after temporarily being constricted it dilates again; this is a known forensic phenomenon.^15–17^ The constriction and dilation of pupils are controlled by the autonomic nerves when alive, similar to the smooth muscles of the airway. If changes in the pupils are caused by discontinuation of neurological functions at death, temporary expansion of the trachea and bronchi could be caused by similar physiological changes. We found no organic changes in the excised trachea and bronchi on pathological analysis 48 hours after death, and although there are no reports regarding the trachea and lung, this result could indicate physiological changes caused by a lack of autonomic nerve control. Fluid from pulmonary edema that develops postmortem infiltrates the alveolar wall and discharges into the trachea and bronchi, which flow back and expand from the peripheral to central airway.^18^

In the present study, both temporal CT images (Figure 4) and pathological findings confirmed an increase in lung field density indicating pulmonary edema and increasing discharge to the peripheral bronchi. The discharge spreading into the trachea and bronchi could be one of the causes of the decreased air content in the trachea and bronchi 48 hours postmortem. Alveolar and airway water clearance are controlled by type II alveolar epithelial cells and bronchial epithelial cells, respectively, via the sodium potassium ATPase and sodium channel.^19–21^ We speculate that cessation of these cell functions after 12 hours postmortem may have led to the results shown in Figure 4.

If similar postmortem expansion of the trachea and bronchi can be verified by imaging analysis of AMCT and PMCT data acquired from humans dying in hospital, this information could be useful to estimate the time of death in cases without organic abnormality of the respiratory system, and in forensic cases where time of death is unknown.

In this study, we used small-animal CT to determine temporal changes in the lung field and attempted to compare these changes with pathological findings following death. However, because the rats were small and we did not perform high-resolution imaging of the lung field, we were unable to assess interstitial changes such as ground-glass opacification.

Identification of the temporal and postmortem changes occurring in the lung field are future challenges. We hope to study many more cases and examine the changes occurring following experimental changes in method of euthanasia, body temperature, body position, rat variety, and gender. Furthermore, we hope to examine the effects of different anesthetic conditions to exclude direct effects of inhalation of anesthetics.

In conclusion, using a rat model, we confirmed that the air content of the lungs decreased after death, whereas the volume of the trachea and bronchi temporarily increased, indicating the potential to use such data to estimate the time of death.
